# Can Inbound Tourism Improve Regional Ecological Efficiency? An Empirical Analysis from China

**DOI:** 10.3390/ijerph191912282

**Published:** 2022-09-27

**Authors:** Liang Zhao, Lifei Xu, Ling Li, Jing Hu, Lin Mu

**Affiliations:** 1 Tourism School, Hubei University, Wuhan 430062, China; 2 School of Tourism and Hospitality Management, Wuhan City Polytechnic, Wuhan 430064, China; 3 Tourism Quality Supervision and Management Institute, Ministry of Culture and Tourism, Beijing 430051, China

**Keywords:** inbound tourism, regional ecological environment, threshold effect

## Abstract

Inbound tourism has an important impact on regional eco-efficiency. This paper uses the panel data of 31 provincial administrative units in China from 2005 to 2019; uses the improved DEA model to measure the regional ecological efficiency; and uses the panel threshold model to investigate input, output, and efficiency from the perspective of green technology innovation. Then, it explores the heterogeneous effects of inbound tourism on ecological efficiency. This paper finds that cross-border tourism has a positive impact on the ecological efficiency of tourist destinations. However, the degree of influence varies and will be changed with the level of regional green innovation. The main conclusions are as follows: (1) From an overall perspective, inbound tourism has a significant positive effect on ecological efficiency. (2) With the increase in green innovation investment and output, the promotion effect of inbound tourism on regional ecological efficiency first increases and then decreases. (3) The higher the green innovation efficiency, the greater the promotion effect of inbound tourism on ecological efficiency. Therefore, the Chinese government should encourage the development of inbound tourism, adopt greener innovative technologies that are cleaner and more environmentally friendly, and enhance the welfare effect of tourism on green economy.

## 1. Introduction

The Industrial Revolution ushered in the path of modern economic growth, and the global economy has become increasingly interconnected [1,2,3]. Even in the context of the emergence of the COVID-19 epidemic, the world has entered a significant change unseen in a century, but globalization has never stopped its pace. Tourism has a global character [4,5,6]. It not only changed people’s living habits and production methods, but also allowed residents to rest mentally and physically, improved their health, and promoted the construction of social industries and infrastructure [7,8,9,10,11]. In practice, tourism is a pillar industry in many developing regions and has a significant and positive role in promoting the production and life of the local people [12,13,14,15,16].

In recent years, “ecological priority, green development” has become China’s national strategy. The Chinese government has clearly proposed accelerating the improvement of economic quality and efficiency and the transformation and upgrading of industrial structure so as to form a green development mode and achieve high-quality development [17,18,19,20,21,22]. As a strategic pillar industry of the national economy, the comprehensive contribution rate of tourism to the national economy and employment has exceeded 10%. According to data from the National Bureau of Statistics, from 1999 to 2018, the number of inbound tourists in China increased from 72.79 million to 145 million, and the foreign exchange income from international tourism increased from US$14.099 billion to approximately US$130 billion [23,24,25]. Inbound tourism has developed steadily. As an essential part of the open economy and modern service industry, compared with domestic tourism, inbound tourism has a higher consumption level and structure, stronger industrial demonstration and related industry driving force, and has cultural soft power shaping and national image output [26,27,28,29].

For a long time, tourism has been shown as a “green industry” and has become an essential carrier of ecological civilization construction and green development [30]. On the one hand, it is attributed to following the tourism-oriented economic growth mechanism, and tourism development brings direct and indirect economic benefits to the destination [31]. On the other hand, compared with the manufacturing industry, the tourism economy consumes less natural capital and emits fewer pollutants [32,33,34,35,36]. It cannot be ignored that the “resource curse” and “Dutch disease effect” that may be caused in the process of inbound tourism development will restrict the green development of the destination [37]. Therefore, under the current practical background, taking inbound tourism as the core observation object and demonstrating whether it promotes the green development of tourism destinations has become a practical question that needs to be answered urgently.

For a long time, academic circles have been concerned about the economic growth and ecological environment of tourism, and fruitful results have been formed. Empirical research on the impact of tourism economic growth, mostly around the hypothesis of tourism-oriented economic growth [38], was performed. Based on the economic paradigm and framework, the hypothesis was verified by using time series data or panel data [39]. On the one hand, research on the environmental effects of tourism is mainly based on the micro-target stratum, geography and ecology paradigm, and framework, and uses field survey measurement or remote sensing data to investigate environmental pollution and ecological problems in the process of tourism development [40,41,42]. On the other hand, scholars have conducted empirical research on the overall pollutant emission of the region affected by tourism development from the macro level [43].

It is not difficult to find that the academic research on the impact of tourism development on destinations takes the destination economic and environmental systems as two independent systems. There is a lack of good interaction between economic impact research and environmental impact research on tourism development. Green development emphasizes the realization of social development and economic growth under the constraints of resources and the environment and has the dual connotation of economic growth and ecological environment protection [44]. To explore the impact of inbound tourism on green development, we can integrate the issues in the above two fields into the same analytical framework for a comprehensive investigation and form a perfect research topic and vision. Referring to the existing research, this paper finds that there are severe limitations in discussing regional environmental problems only from the perspective of environmental pollution because the operation of the economy and society is behind environmental pollution [45,46,47]. Therefore, this paper chooses ecological efficiency as a quantitative method to measure the level of green development in the region and examines the impact of inbound tourism on ecological efficiency.

It is worth noting that the improvement of ecological efficiency is inseparable from the contribution of green innovation. With the current climate warming and environmental problems escalating, it is imperative to integrate ecological and environmental protection into the overall situation of economic development [48]. Green technology innovation has also become an important factor in balancing economic growth and environmental protection. At the same time, green technology innovation is an important driving force for enterprises to improve their own competitive advantages, and it is also a meaningful way to reduce carbon emissions and improve environmental pollution [49,50,51,52]. Therefore, under the different levels of green innovation in different regions, the impact of inbound tourism on ecological efficiency will inevitably produce heterogeneity. Therefore, this paper will apply the panel threshold model to study the role of heterogeneity from the perspectives of green innovation input, green innovation output, and green innovation efficiency. The structure of the paper is as follows: the second part is the theoretical hypothesis and literature review; the third part is the selection of data and the establishment of the empirical model; the fourth part is the empirical model and results analysis, including the benchmark regression model and threshold regression model; the fifth part is conclusions and policy recommendations.

## 2. Literature Review and Theoretical Hypotheses

The international development of tourism is closely related to regional economic growth. When more and more people enter the Chinese tourism market, tourism regions will receive a large amount of foreign exchange income, which can achieve regional economic growth. If people use this part of the income to improve the tourism industry and equipment, it is bound to form a virtuous circle in the development of the clean tourism industry. When people from different regions, nationalities, and cultural backgrounds communicate with each other in the process of tourism, it is conducive to promoting the collision and integration of cultures and improving the diversity of cultures [53,54,55,56].

First, based on understanding ecological efficiency and its growth source, this paper analyzes the mechanism of inbound tourism on the basis of ecological efficiency. Upgrading should be discussed at two levels of environmental inputs, and the production of final goods: on the one hand, from the perspective of environmental investment: (1) inbound tourism needs the support of good regional transportation conditions. The construction and coverage of modern transportation networks such as airports and high-speed rail will strengthen the cooperation between the destination and surrounding cities and form a good location for economic development so as to improve the regional economic efficiency [57,58,59]. (2) Inbound tourism not only promotes the construction of infrastructure and hardware at the destination but also promotes software changes such as management systems and policies, thereby improving the city’s overall economic development environment and optimizing resource allocation efficiency. (3) Inbound tourism may bring advanced production technology and management experience by promoting foreign direct investment in the opening of destinations and improving production efficiency [60,61,62,63,64]. (4) The improvement of scale efficiency is one of the ways to improve ecological efficiency. With the gradual improvement of basic supporting facilities, the application and promotion of new production technologies, and the good development of related industries and new business forms of industrial integration, it will help to achieve the scale effect of production at the destination [65,66,67].

On the other hand, from the perspective of the production of final goods: (1) The tourism development will promote urban innovation capabilities. Inbound tourism triggers innovative behaviors through the integration of related industries such as leisure agriculture, industrial tourism, cultural tourism, tourism real estate, and smart tourism [68,69,70,71,72]. (2) Inbound tourism interferes with the changes in the industrial structure of the destination. The development of inbound tourism depends on the good ecological environment of the destination, which will prompt the government to implement stricter environmental regulation policies and increase the supervision of energy consumption and pollutant emissions. Low-value-added and high-consumption industries are squeezed out, forcing the destination industry to adjust its industrial structure. The advanced and clean industrial structure is bound to improve the destination technology level and the reduction in undesired output [73,74,75]. (3) Inbound tourism promotes the opening of the destination to the outside world and promotes the destination to form a good reputation and reputation, which is conducive to attracting foreign direct investment, thereby developing high-tech industries and promoting the improvement of the city’s comprehensive innovation capability [76]. In summary, this paper proposes the first hypothesis:

**Hypothesis** **1** **(H1).** *Inbound tourism is beneficial to improve regional ecological efficiency*.

Green innovation refers to a new type of innovation activity that combines the dual interests of technological innovation and environmental protection to ease the pressure on resources and the environment while increasing economic benefits. Green innovation requires a cross-regional flow of innovative elements such as green knowledge and technology for broad participation [77]. Therefore, green innovation helps us to further explain the theoretical basis of the nonlinear mechanism of inbound tourism affecting ecological efficiency. From the perspective of green innovation input and output, inbound tourism may have a heterogeneous impact on ecological efficiency. The reasons are as follows: Combined with new economic geography, it can be seen that central cities often have superior location conditions, with the development advantages of large market scale and excellent infrastructure, and can attract more innovation elements to flow in and gather, thus, contributing to the formation of a suitable environment for green innovation and development. On the contrary, the location conditions of non-central cities are generally inferior to those of central cities, and their attractiveness to innovation elements is relatively weak, which is not conducive to developing urban green innovation. In the case of uneven regional development, the central city usually has a dual effect of “diffusion” and “siphoning” on the peripheral cities. Therefore, cities with more green innovation resources can often produce agglomeration effects and scale effects, which are conducive to improving regional ecological efficiency [78].

However, from another perspective, the excessive concentration of green innovation elements will have a crowding effect, and the ecological environment cannot support excessive tourism development [79]. On the one hand, the quality of innovation elements varies, and too much element agglomeration will inevitably lead to difficult choices for enterprises and non-market-oriented behaviors such as collusion [80]. On the other hand, too many innovative elements will also lead to uneven distribution and mismatch of elements [81]. Therefore, the agglomeration of innovative elements within a specific region may negatively impact inbound tourism on ecological efficiency. In summary, this paper proposes the second hypothesis:

**Hypothesis** **2** **(H2).** *Differences in the number of innovation factors may impact the relationship between inbound tourism and ecological efficiency*.

Green innovation efficiency refers to the input–output ratio of valuable creative activities that promote the development of green technology under the constraints of no pollution, low energy consumption, and cleanliness. Previous studies on the impact of environmental regulation on the efficiency of green innovation are mainly divided into two theoretical foundations [82]. To create a good regional ecological environment, the more developed the tourism industry, the stricter the environmental regulations tend to be. According to the relevant theories of environmental regulation, one is the “Innovation Compensation Theory” represented by Porter, which means that strict environmental regulations will force enterprises to carry out green technology innovation, thereby improving the ecological efficiency of the entire region. And the other is the “Following Cost Theory”. It means that strict environmental regulation will increase enterprises’ production cost, reduce enterprises’ production efficiency, and may negatively impact the regional ecological efficiency [83]. Therefore, this paper proposes the third hypothesis:

**Hypothesis** **3** **(H3).** *With the improvement of green innovation efficiency, the impact of inbound tourism on ecological efficiency is uncertain, and there may be a threshold effect*.

## 3. Model Construction and Data Selection

### 3.1. Model Construction

#### 3.1.1. Benchmark Regression

To verify the relationship between inbound tourism and ecological efficiency, this paper establishes a panel least squares regression model:

(1) $$Y_{\mathrm{it}}{=\beta }_{1}X_{\mathrm{it}}+\beta Control_{\mathrm{it}}{+\beta }_{0}{+\varepsilon }_{\mathrm{it}}+i+t$$

Among them, *Y* is the explained variable, that is, ecological efficiency; *X* is the explanatory variable, that is, the number of inbound tourists; and *Control* is the control variable. After the Hausman test, this paper adopts the time-region fixed effect model. Therefore, *t* is the time fixed effect; *i* is the individual fixed effect; $\varepsilon_{\mathrm{it}}$ is the random interference item; *β*, *β*_0_, *β*_1_ are the coefficients to be estimated.

#### 3.1.2. Panel Threshold Model

The threshold regression developed by Hansen (1999) [84] tests whether the parameters of the sample groups divided according to the threshold value are significantly different, and it is used to study the heterogeneity of the interaction between variables. Under the accumulation of different innovative elements, there may be a nonlinear relationship between inbound tourism and ecological efficiency, resulting in the results of threshold model regression being more realistic. Considering the possible spatial correlation of ecological efficiency, first, the following spatial panel threshold model is set:

(2) $$Y_{\mathrm{it}}{=\lambda }_{0}{+\lambda }_{1}D_{\mathrm{it}}{\cdot I(\mathrm{thre}}_{\mathrm{it}}\leq r_{1}{)+\lambda }_{2}D_{\mathrm{it}}{\cdot I(\mathrm{thre}}_{\mathrm{it}}{>r}_{1}{)+\lambda }_{3}X_{\mathrm{it}}{+\gamma \cdot t+\varepsilon }_{\mathrm{it}}\;$$

where I(⋅) represents the indicative function, which takes the value 1 when the expression in the parentheses is true and 0 when it is false. *D_it_* is the core explanatory variable, w*D_it_ refers to the spatial lag term of the explained variable, ${\mathrm{thre}}_{\mathrm{it}}$ is the threshold variable, *X_it_* is the control variable, *ε_it_* is the random disturbance term. When ${\mathrm{thre}}_{\mathrm{it}}$ ≤ *r*_1_, the core explanatory variable *D_it_* coefficient is *λ*_1_, when ${\mathrm{thre}}_{\mathrm{it}}$ > *r*_1_, the core explanatory variable *D_it_* coefficient is *λ*_2_, t is the time effect, *λ* is a constant term, *ε_it_*~(0, *σ*) is a random interference term. The similarities and differences between *λ*_1_ and *λ*_2_ are what we focus on.

Equation (2) only assumes one threshold, but there may be two or more thresholds. Due to space limitations, the test for two or more thresholds will not be repeated here.

### 3.2. Indicator Construction

#### 3.2.1. Explained Variable (Ecological Efficiency, Ecoe)

Ecological efficiency is a productivity analysis of input and output, which is suitable for evaluation by data envelopment analysis (DEA), that integrates linear programming and multi-objective programming. Since the traditional DEA model cannot further accurately divide the effective decision-making units, this paper adopts the improved DEA model [85] to measure the ecological efficiency of the region:

There are *n* decision-making units *DMU*_k_, (k = 1, 2…*n*), each decision-making unit has *m* input index and *s* output index, the input vector is *X_k_* = (*X*_1*k*_, …, *X_mk_*)^T^, and the output vector is *Y_k_* = (*Y*_1*k*_, …, *Y*_s*k*_)^T^. Among them, *x_ik_* and *y_rk_* represent the *i*th input index value and the *r*th output index value of *DMU_k_*, respectively, *v_i_* and *u_r_* are the weight coefficients of the corresponding indexes, respectively; *C_m_* and *B_s_* are the judgments constructed according to the importance of the input indexes and output indexes. Matrix; *λ_m_* and *λ_s_* are the maximum eigenvalues of the judgment matrices *C_m_* and *B_s_*, respectively.

On the basis of the existing *DMU*, two virtual *DMUs* are introduced, namely, the optimal *DMU* and the worst *DMU*, which are respectively recorded as *DMU_n+_*_1_ and *DMU_n+_*_2_. The input index value of the optimal virtual decision-making unit *DMU_n+_*_1_ takes the minimum value of the corresponding index values of *n* actual *DMUs*, and the output index value takes the maximum value of the corresponding index values of *n* actual *DMUs*; similarly, the worst virtual decision-making unit *DMU_n+_*_2_*_,_* the input index value of *n* takes the maximum value of the corresponding index values of *n* actual *DMUs,* and the output index value takes the minimum value of the corresponding index values of *n* actual *DMUs*. The specific form of the improved DEA model is shown in formula (3).

(3) $$\{\begin{array}{l} \min\sum_{r=1}^{s}u_{r}y_{r,n+2}\\ s.t.\sum_{i=1}^{m}v_{i}x_{i,n+2}=1\\ \begin{array}{cc} & \sum_{r=1}^{s}u_{r}y_{r,n+1}-\sum_{i=1}^{m}v_{i}x_{i,n+1}=0 \end{array}\\ \begin{array}{cc} & \sum_{r=1}^{s}u_{r}y_{rj}-\sum_{i=1}^{m}v_{i}x_{ij}\leq 0\begin{array}{cc} , & j\neq n+1 \end{array} \end{array}\\ \begin{array}{cc} & (C_{m}-\lambda_{m}E_{m})v\geq 0 \end{array}\\ \begin{array}{cc} & (B_{s}-\lambda_{s}E_{s})u\geq 0 \end{array}\\ \begin{array}{cc} & u_{r}\geq 0\begin{array}{cc} , & r=1,2,\cdots ,s \end{array} \end{array}\\ \begin{array}{cc} & v_{i}\geq 0\begin{array}{cc} , & i=1,2,\cdots ,m \end{array} \end{array} \end{array}$$

The public weights are obtained from the above model ur*, vr*, use the formula:

(4) $$\theta_{k}^{*}=\sum_{i=1}^{s}u_{r}^{*}y_{rk}/\sum_{i=1}^{m}v_{{}_{i}}^{*}x_{ik}$$

**Figure 1:**
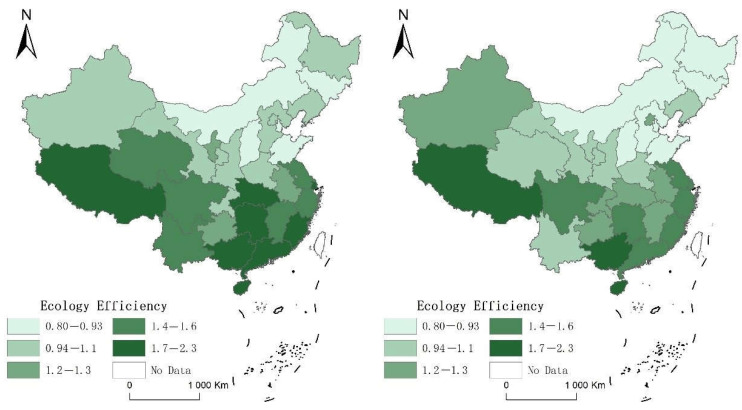
The spatiotemporal evolution pattern of ecological efficiency.

Calculate the relative efficiency value of each *DMU*. The larger the value, the higher the system operating efficiency. It can be seen that to apply the above method to calculate the urban ecological efficiency, it is also necessary to determine the input and output of ecological efficiency. In this paper, natural resources input and economic factor input are used as input variables, and the two dimensions of economic expected output and ecological environment load are used as output variables to examine ecological efficiency. The specific indicators are constructed as follows (Table 1):

Figure 1 shows the spatiotemporal evolution and spatial differentiation of ecological efficiency, in which the left picture is 2005, and the right picture is 2019. It can be found that China’s overall ecological efficiency is at a declining level. The reason is that the added value of China’s manufacturing industry continues to increase, and environment-intensive and pollution-intensive enterprises have been the most critical factors driving economic growth in the past decade. With the rapid development of China’s economy, the situation of tightening resource constraints, serious environmental pollution, and ecosystem degradation has become increasingly severe, and the long-term economic development model of “high investment, high consumption, and high emissions” has led to the development of regional resources, environment, and economy. The contradiction between them is becoming more and more prominent. From the perspective of sub-regions, it can be found that the ecological efficiency of the central region declines the fastest because the central region is the transition zone of China’s economy. Although the resource abundance is higher than that of the eastern region, its economic foundation and scale are far inferior to those of the eastern region. Its resource utilization efficiency and the capital conversion rate are low, so the evolution of ecological efficiency is below the medium level.

#### 3.2.2. Explanatory Variables (Ibtr)

The main reason is that inbound tourism is an economic activity; the impact of inbound tourism on the ecological environment is transmitted through economic behavior, and the impact of inbound tourism on green innovation behavior also requires incentives from economic behavior. Therefore, this paper uses inbound tourism revenue to measure the city’s inbound tourism development level.

#### 3.2.3. Threshold Variables

This paper uses green innovation input (Grii), green innovation output (Grio), and green innovation efficiency (Grie) as threshold variables [86]. This paper uses human resources and green capital as input variables of green innovation; selects green patents; the number of scientific research papers on the green environment as output variables of green innovation. At the same time, the improved DEA model is used to calculate the efficiency of urban green innovation [87].

#### 3.2.4. Control Variable

This paper selects control variables from social, environmental, and economic variables that affect ecological efficiency [88,89]. Opening up (Fdi), measured by foreign direct investment; economic level (Econ), measured by per capita GDP; industrial structure (Ind), measured by the proportion of tertiary industry in GDP; environmental regulation intensity (Envi), removal of industrial soot and urbanization (Urb), measured by the proportion of construction land in the total area; innovation capability (Crea), measured by the proportion of science and technology expenditure in GDP.

### 3.3. Data Sources

The patent data in this article come from the CNKI patent database, and the rest of the variables come from the Wande database, “China Statistical Yearbook of Population and Employment”, “China Statistical Yearbook”, “China Environmental Statistical Yearbook”, “China Agricultural Yearbook”, “China Land and Resources Statistical Yearbook”, and “China Urban Statistical Yearbook”; the time interval is 2005–2019. The reason for choosing 2019 as the time node is that the outbreak of the COVID-19 epidemic in 2020 had a great impact on economic and social indicators; the reason why 2005 is chosen as the starting point is that before 2004, there were a large number of county withdrawals in China. The adjustment of administrative divisions such as districts is likely to have a relatively adverse impact on the quality of data.

## 4. Empirical Results and Analysis

### 4.1. Benchmark Regression Results

**Figure 2:**
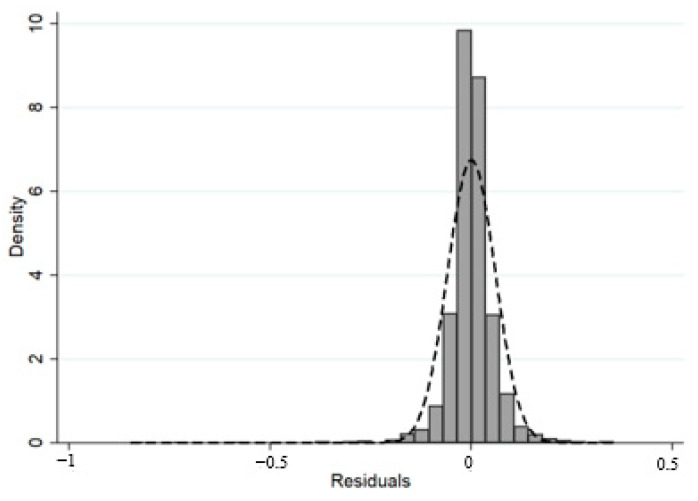
Residual distribution diagram.

Before conducting the empirical analysis, this paper conducts a normality test to ensure that the residuals conform to a normal distribution. If the classical assumption is violated, the parameter estimates will not have the minimum variance, that is, the validity will be lost; if the assumption of normality is violated, the *t* statistic will not obey the *t* distribution, and the *t*-test will fail. The normality test tests whether the assumption that the residuals follow a normal distribution with zero mean is true. This paper uses the Shapiro–Wilk test and the Shapiro–Francia *W*-test to verify the normality of the residuals. After testing, the assumption that the residuals do not obey the normal distribution is rejected (Figure 2).

It can be found that the promotion effect of inbound tourism on ecological efficiency exists at the 1% significance level, and each unit of inbound tourism indicator increases ecological efficiency by 0.3 units. The promotion of ecological efficiency by inbound tourism means that inbound tourism can stimulate innovative behaviors of new business forms based on industrial integration and introduce advanced technologies and innovation capabilities through the open economy to promote the creativity of the destination. Its practical policy direction is obvious. The development of inbound tourism is a reliable policy tool to promote regional green development, and the effectiveness of this tool is related to the economic level of the destination; that is to say, a good urban development background can better play a role in inbound tourism. The green development effect provides external conditions. The destination should take “city-tourism integration” as the core and practice the concept of global tourism development: on the one hand, inbound tourism should drive and promote the coordinated economic and social development and the optimization and improvement of the destination city system; on the other hand, the overall planning layout and overall coordination management in the process of economic development of the destination should take into account the development demands of inbound tourism. The achievements of economic construction are shared with the tourism industry, forming a positive interaction pattern between inbound tourism and the city’s overall economic development. Additionally, it will finally realize the green development effect of inbound tourism.

For the control variables, it can be found that: (1) With the improvement of the opening up of the region, the region’s ecological efficiency is also constantly improving. The reason may be that the opening to the outside world is conducive to clean production and production of developed countries with high technology. Green innovation technology can spill over to developing countries to a certain extent; on the other hand, developing countries are more inclined to introduce cleaner production technology to achieve economic and environmental benefits. (2) The improvement of the regional economic development level will reduce the ecological efficiency. The reason is that for China, its GDP structure is still dominated by high-polluting industries, so the development of the economy will bring environmental pollution to a certain extent. (3) The proportion of the tertiary industry in GDP can significantly improve the regional ecological efficiency, which verifies that the development of tourism will reduce environmental pollution. (4) The intensity of environmental regulation helps to improve ecological efficiency. Different cities have different development stages and strategies, and there are differences in the level of green development and the intensity of environmental regulation. Some of the more developed cities or those facing greater resource and environmental pressures took the lead in changing their development methods to achieve the dual improvement of innovation efficiency and environmental benefits. Other cities can gain experience in their green innovation development, thereby achieving an overall improvement in the efficiency of green innovation in all cities (Table 2).

### 4.2. Threshold Regression Results

#### 4.2.1. From the Perspective of Green Innovation Factor Input

Green technology innovation is an indispensable part of the green transformation process of enterprises. Compared with pollution control and emission reduction achieved by limiting its traditional production capacity, green technology innovation can create economic value and reach a win–win situation in environmental performance and corporate performance. The input of green innovation elements can be reflected by selecting the number of scientific and technological employees of environmental protection enterprises in urban units and the scientific and technological expenditure of environmental protection public finance from the two aspects of human resources and capital. With the different scales of green innovation elements, there will be some differences in the promotion of inbound tourism to ecological efficiency. The reason is that the agglomeration of innovative elements deepens the connection between enterprises, and enterprises’ “green washing” behavior will be quickly communicated to other enterprises and financial institutions. In this way, low-quality enterprises will not dare to act lightly due to high default costs, further mitigating information asymmetry’s negative impact. Therefore, the information conveyed by the green technology innovation of enterprises within the agglomeration will be more credible.

Table 3 shows the test of the threshold effect, referring to the existing literature, we selected 300 times of BS. It was found that whether it was a single-threshold, double-threshold, or three-threshold model, the threshold effect exists at the 10% significance level. 

Table 4 shows the regression results of the threshold effect. The threshold values selected in this paper are presented as percentages. For example, the single-threshold model, the variables below the threshold value account for 14.2% of the sample. The double-threshold model, the samples between the first and second thresholds accounted for 18.4% of the total.

From the single-threshold model, it can be found that when the green innovation factors are invested in the transnational threshold, the promoting effect of inbound tourism on ecological efficiency increases; however, the double-threshold and three-threshold models tell us that with the increase in the number of green innovation factors, the promotion effect of inbound tourism on ecological efficiency shows a characteristic of increasing first and then decreasing. For the control variable, it can be found that the positive and negative coefficients and the significance are similar to the benchmark regression part, and this article will not repeat them.

#### 4.2.2. From the Perspective of Green Innovation Factor Output

The output of green innovation mainly includes papers and patents related to “ecological environment”, “clean production”, “sustainable development”, and “carbon emission”. Different from general technological innovation, green innovation has strong technical attributes and spillover characteristics from R&D innovation. The specific performance is the dual externality effects of the innovation and diffusion stages. This effect will benefit the society or other enterprises, while the enterprises engaged in green innovation will bear higher innovation costs. Therefore, the promotion of green innovation may affect the impact of inbound tourism on ecological efficiency [90]. Table 5 shows the threshold effect test of green innovation output as a threshold variable. It can be found that the threshold models of three different categories are all established at the 1% significance level. Therefore, the following three models will be analyzed separately.

Table 6 shows the regression results of the threshold effect. From the single threshold model, it can be found that with the increase in green innovation output, the impact of inbound tourism on ecological efficiency changes from strong to weak. However, from the double-threshold and triple-threshold models, it can be found that this effect first decreases and then increases with the increase in green innovation output. The reason is that green innovation has a specific cost. The dual externalities of green innovation and the spillover of R&D innovation will inhibit the enthusiasm of new energy companies to invest in green R&D [91]. Therefore, government policy guidance and government subsidies become very necessary. Under the conditions of market failure or imperfect market mechanism, financial subsidies use the form of price subsidies to alleviate and offset the unreasonable price structure or eliminate the adverse effects of exclusionary effects. However, in the initial stage of green innovation, government subsidies can play a significant role. However, with green innovation’s deepening, enterprise viability has become very important [92]. Therefore, increasing green innovation output will bring a significant scale effect, which will help enterprises to generate self-viability. Then, the scale effect will appear after the green innovation output has accumulated to a certain extent. This will contribute to the promotion of the ecological efficiency of inbound tourism.

#### 4.2.3. From the Perspective of Green Innovation Efficiency

A free-market environment gives high-tech industries more opportunities to develop a green economy. With the globalization of the economy, under the regulation of the market mechanism, industries with high pollution and resource consumption will be gradually eliminated, and pollutant emissions will be reduced in sustainable development. However, due to differences in resource endowments and stages and levels of economic growth, there are significant differences in the innovation capabilities and green development levels of cities and the efficiency of green innovation. Therefore, this heterogeneity will significantly change the depth and breadth of the potential impact of inbound tourism on ecological efficiency. Table 7 shows the test results of the threshold effect. Again, all three models are significantly established.

Table 8 shows the regression results of the threshold effect. It can be found that with the improvement of green innovation efficiency, the promotion effect of inbound tourism on ecological efficiency has been continuously enhanced. The reason is that efficiency is an indicator that considers both input and output dimensions. Therefore, improving efficiency will inevitably bring positive externalities, which will contribute to the heterogeneous effect of inbound tourism on ecological efficiency. Thus, at present, China urgently needs to carry out “green” and “innovative” production activities to improve economic quality.

## 5. Conclusions and Policy Recommendations

At this stage, the discussion on the green attributes of the tourism industry remains at the level of whether the tourism industry itself is green, but in view of the comprehensive driving force of the tourism industry, it will become more meaningful to examine the “externality” of the tourism industry to the green development of the destination. Therefore, incorporating inbound tourism and destination green development into the same analytical framework reveals the impact of inbound tourism on regional ecological efficiency from three perspectives: green innovation input, green innovation output, and green innovation efficiency. The final empirical results support the conclusion that inbound tourism is a green industry driving regional green development, and it is found that the green development effect of inbound tourism has nonlinear characteristics with the level of green innovation as the threshold variable. The main conclusions drawn from this paper are as follows.

### 5.1. Conclusions

(1) Inbound tourism can significantly improve regional ecological efficiency: the global environment has profoundly changed since the Industrial Revolution. Ecosystems in ecologically fragile areas have poor stability, weak anti-interference, and self-recovery capabilities. Under the background of global change, natural resource supply capacity declines, land degradation, biodiversity reduction, and frequent disasters. Ecosystems face enormous risks. Therefore, countries should use inbound tourism as a driving force to improve regional ecological efficiency and ecological security.
(2) With the increase in green innovation investment, the promotion effect of inbound tourism on regional ecological efficiency first increases and then decreases. Enterprises are the source of innovation. In fact, excessive R&D investment is undeniable in the business practice of enterprises, and information asymmetry will exacerbate the moral hazard of management’s opportunistic behavior. If the company’s innovation investment opportunities and investment benefits are symmetrical between shareholders and managers, managers’ over-investment or under-investment in innovative projects will be observed by shareholders, and shareholders will take measures to avoid losses to reduce agency costs.
(3) With the improvement of green innovation output, the promotion effect of inbound tourism on regional ecological efficiency first decreases and then increases. It can be found that for a region, its innovation bottleneck still exists. From experience and facts, some regions are in trouble because they cannot successfully overcome some systemic bottlenecks in the process of modernization. For example, economic development cannot transform into an innovative economy, and they fall into the “middle-income trap”. This will be detrimental to the improvement of regional ecological efficiency.
(4) The higher the efficiency of green innovation, the greater the promotion effect of inbound tourism on ecological efficiency. It can be found that green innovation efficiency is vital in promoting ecological efficiency. Therefore, it is necessary to speed up the improvement of urban green innovation efficiency, form a mutual promotion mechanism of innovation drive and green development, and realize the synergy and win–win of technological progress, green ecology, and economic benefits.

### 5.2. Policy Recommendations

The above research conclusions provide theoretical support for the green industry attributes of inbound tourism in the context of China’s practice and have positive practical policy implications: First, no matter what stage of development the destination economy is in, from the perspective of promoting the overall green development of the city, local governments should make full use of their own resource characteristics and location advantages and give stronger policy support to strengthen opening to the outside world. The development of inbound tourism should be encouraged and used as an effective tool to achieve urban green development. Second, a sound urban development foundation will provide favorable external conditions for inbound tourism to promote the green development of destinations. Therefore, to give full play to the green development effect of inbound tourism, the development of inbound tourism should be placed in the overall layout of urban economic improvement. By promoting the integrated development of urban tourism, a pattern of positive interaction and mutual promotion between the development of inbound tourism and the economic development of the destination will be formed.

## Figures and Tables

**Table 1:** Ecological efficiency input–output variables.

Criterion Layer	Indicator Layer	Specific Instructions
Natural resource inputs	Water input	Total urban water consumption/10^4^ cubic meters
	Energy input	Total urban electricity consumption/10^4^ kWh
	Land input	Urban construction land area/square kilometer
Input of economic factors	Labor input	Number of employees in the unit/10^4^ people
	Capital investment	Fixed asset investment/10^4^ yuan
Economic expected output	Regional GDP	Regional GDP/10^4^ yuan
Ecological load	Water pollution	Discharge of industrial wastewater/10^4^ tons
	Air Pollution	Industrial sulfur dioxide emissions/10^4^ tons

**Table 2:** Benchmark regression results.

	(1) OLS	(2) FE	(3) D-K	(4) GMM	(5) Drop Variable	(6) 2SLS
*Ibtr*	0.27 ***	0.31 ***	0.35 ***	0.31 ***	0.36 ***	0.31 ***
	(4.08)	(2.82)	(3.98)	(3.29)	(3.49)	(4.08)
*Fdi*	0.22 ***	0.20 ***	0.22 ***	0.19 ***	0.20 ***	0.29 ***
	(4.54)	(3.64)	(4.53)	(3.77)	(3.18)	(3.15)
*Econ*	−0.22 ***	−0.22 ***	−0.21 ***	−0.21 ***	−0.17 ***	−0.21 ***
	(−3.97)	(−4.46)	(−4.55)	(−3.40)	(−3.13)	(−2.83)
*Ind*	1.74 ***	2.50 ***	1.87 ***	2.33 ***	2.49 ***	1.70 ***
	(3.45)	(3.09)	(3.37)	(3.24)	(2.97)	(3.78)
*Envi*	2.25 **	2.95 **	2.43 *	2.96 ***	2.70 *	2.30 ***
	(2.32)	(2.22)	(1.97)	(1.93)	(1.77)	(2.74)
*Urb*	−0.13	−0.09	−0.09	−0.13	−0.09	−0.11
	(−0.93)	(−1.22)	(−1.34)	(−0.87)	(−1.30)	(−1.04)
*Crea*	0.09	0.08	0.10	0.11	0.09	0.10
	(0.36)	(0.36)	0.43	0.46	0.37	(0.50)
Time*Individual *fixed effects*	Control	Control	Control	Control	Control	Control
*Cons*	1.20 ***	1.17 ***	1.06 ***	1.13 ***	1.01 ***	1.09 ***
	(3.42)	(3.32)	(4.92)	(3.72)	(4.43)	(3.97)
*R* ^2^	0.6129	0.6251	0.6293	0.6228	0.5711	0.7014
*Obs*	450	450	450	390	415	450

Note: *t* values are in parentheses, ***, **, and * indicate significance at the 1%, 5%, and 10% levels, respectively.

**Table 3:** Threshold effect test: From the perspective of green innovation factor input.

	F Value	*p* Value	1% Critical Value	5% Critical Value	10% Critical Value
Single-threshold	3.321 *	0.093	7.706	4.932	3.249
Double-threshold	2.359 *	0.073	8.271	2.955	2.054
Three-thresholds	9.062 ***	0.000	3.954	2.252	1.633

Note: *** and * indicate significance at the 1% and 10% levels, respectively.

**Table 4:** Threshold effect results: From the perspective of green innovation factor input.

		(1) Single-Threshold	(2) Double-Threshold	(3) Three-Thresholds
*Ibtr*	*Grii* < *δ*_1_	0.25 ***	0.27 ***	0.30 ***
		(3.61)	(2.47)	(3.31)
	*δ*_1_ ≤ *Grii* < *δ*_2_	0.34 ***	0.37 ***	0.35 ***
		(3.79)	(4.08)	(3.74)
	*δ*_2_ ≤ *Grii* < *δ*_3_		0.30 ***	0.28 ***
			(3.08)	(3.22)
	*δ*_3_ < *Grii*			0.27 ***
				(2.93)
*Fdi*		0.19 ***	0.28 ***	0.26 ***
		(4.88)	(4.64)	(4.66)
*Econ*		−0.17 ***	−0.20 ***	−0.21 ***
		(−3.34)	(−4.37)	(−3.29)
*Ind*		1.58 ***	2.32 ***	2.17 ***
		(4.41)	(3.07)	(4.37)
*Envi*		2.65 ***	3.01 **	2.35 **
		(2.29)	(2.59)	(2.27)
*Urb*		−0.09	−0.12	−0.09
		(−0.98)	(−1.15)	(−1.15)
*Crea*		0.11	0.08	0.10
		(0.53)	(0.50)	(0.37)
*Time effects*		Control	Control	Control
*Individual effects*		Control	Control	Control
*Cons*		0.97 ***	1.10 ***	0.93 ***
		(3.20)	(3.42)	(5.17)
*R* ^2^		0.6106	0.6202	0.6274
*Obs*		450	450	450
*δ*_1_		0.142	0.171	0.322
*δ*_2_			0.355	0.435
*δ*_3_				0.812

Note: *t* values are in parentheses, *** and ** indicate significance at the 1% and 5% levels, respectively.

**Table 5:** Threshold effect test: From the perspective of green innovation factor output.

	F Value	*p* Value	1% Critical Value	5% Critical Value	10% Critical Value
Single-threshold	27.560 ***	0.003	23.971	10.066	2.187
Double-threshold	8.659 ***	0.000	4.728	2.791	1.936
Three-thresholds	24.743 ***	0.007	19.746	6.338	0.059

Note: *** indicate significance at the 1% levels.

**Table 6:** Threshold effect results: From the perspective of green innovation factor output.

		(1) Single-Threshold	(2) Double-Threshold	(3) Three-Thresholds
*Ibtr*	*Grii* < *δ*_1_	0.31 ***	0.31 ***	0.36 ***
		(3.85)	(2.87)	(3.50)
	*δ*_1_ ≤ *Grii* < *δ*_2_	0.28 ***	0.30 ***	0.20 ***
		(3.23)	(3.25)	(3.42)
	*δ*_2_ ≤ *Grii* < *δ*_3_		0.33 ***	0.32 ***
			(2.82)	(4.10)
	*δ*_3_ < *Grii*			0.33 **
				(2.46)
*Fdi*		0.18 ***	0.23 ***	0.23 ***
		(3.23)	(4.84)	(4.67)
*Econ*		−0.19 ***	−0.14 ***	−0.22 ***
		(−4.33)	(−2.93)	(−3.52)
*Ind*		1.67 ***	1.90 ***	2.52 ***
		(4.18)	(2.82)	(3.93)
*Envi*		2.94 **	2.08 **	2.79 **
		(2.41)	(2.29)	(2.33)
*Urb*		−0.11	−0.13	−0.09
		(−1.17)	(−1.14)	(−1.16)
*Crea*		0.10	0.07	0.10
		(0.50)	(0.34)	(0.48)
*Time effects*		Control	Control	Control
*Individual effects*		Control	Control	Control
*Cons*		1.24 ***	1.23 ***	0.87 ***
		(4.56)	(4.92)	(3.86)
*R* ^2^		0.5629	0.5927	0.5998
*Obs*		450	450	450
*δ*_1_		0.468	0.468	0.261
*δ*_2_			0.632	0.621
*δ*_3_				0.897

Note: *t* values are in parentheses, *** and ** indicate significance at the 1% and 5% levels, respectively.

**Table 7:** Threshold effect test: From the perspective of green innovation efficiency.

	F Value	*p* Value	1% Critical Value	5% Critical Value	10% Critical Value
Single-threshold	9.124 ***	0.000	4.329	2.367	1.717
Double-threshold	22.719 ***	0.000	17.085	3.815	0.472
Three-thresholds	34.704 ***	0.000	19.827	13.444	9.024

Note: *** indicate significance at the 1% levels.

**Table 8:** Threshold effect results: From the perspective of green innovation efficiency.

		(1) Single-Threshold	(2) Double-Threshold	(3) Three-Thresholds
*Ibtr*	*Grii* < *δ*_1_	0.22 ***	0.26 **	0.22 ***
		(3.25)	(2.52)	(4.05)
	*δ*_1_ ≤ *Grii* < *δ*_2_	0.42 ***	0.33 ***	0.36 ***
		(2.69)	(2.67)	(3.20)
	*δ*_2_ ≤ *Grii* < *δ*_3_		0.54 ***	0.43 ***
			(2.92)	(3.83)
	*δ*_3_ < *Grii*			0.56 ***
				(3.15)
*Fdi*		0.22 ***	0.19 ***	0.21 ***
		(3.40)	(3.97)	(3.36)
*Econ*		−0.20 ***	−0.19 ***	−0.15 ***
		(−2.89)	(−3.19)	(−4.20)
*Ind*		2.43 ***	2.40 ***	2.27 ***
		(3.14)	(4.03)	(4.48)
*Envi*		3.04 **	3.07 ***	3.03 ***
		(2.29)	(2.98)	(3.10)
*Urb*		−0.11	−0.12	−0.11
		(−1.13)	(−1.21)	(−0.89)
*Crea*		0.07	0.09	0.11
		(0.38)	(0.39)	(0.45)
*Time effects*		Control	Control	Control
*Individual effects*		Control	Control	Control
*Cons*		1.41 ***	0.91 ***	1.08 ***
		(4.14)	(4.74)	(3.11)
*R* ^2^		0.6710	0.6722	0.6771
*Obs*		450	450	450
*δ*_1_		0.277	0.336	0.351
*δ*_2_			0.473	0.688
*δ*_3_				0.864

Note: *t* values are in parentheses, *** and ** indicate significance at the 1% and 5% levels, respectively.

## Data Availability

No new data were created or analyzed in this study. Data sharing is not applicable to this article.
